# Automatic brain quantification in children with unilateral cerebral palsy

**DOI:** 10.3389/fnins.2025.1540480

**Published:** 2025-03-10

**Authors:** Jaime Simarro, Thibo Billiet, Thanh Vân Phan, Simon Van Eyndhoven, Monica Crotti, Lize Kleeren, Lisa Mailleux, Nofar Ben Itzhak, Diana M. Sima, Els Ortibus, Ahmed M. Radwan

**Affiliations:** ^1^icometrix, Leuven, Belgium; ^2^KU Leuven, Department of Development and Regeneration, Locomotor and Neurological Disorders Group, Leuven, Belgium; ^3^KU Leuven, Department of Neurosciences, Leuven Brain Institute, Leuven, Belgium; ^4^KU Leuven, Child and Youth Institute, Leuven, Belgium; ^5^KU Leuven, Leuven Brain Institute, Leuven, Belgium; ^6^KU Leuven, Department of Rehabilitation Sciences, Leuven, Belgium; ^7^Hasselt University, Rehabilitation Research Centre, Faculty of Rehabilitation Sciences, Diepenbeek, Belgium; ^8^UZ Leuven, Department of Pediatric Neurology, Leuven, Belgium; ^9^KU Leuven, Translational MRI, Department of Imaging and Pathology, Leuven, Belgium

**Keywords:** brain volume quantification, neurodevelopmental disorders, cerebral palsy, deep learning, magnetic resonance imaging

## Abstract

Assessing brain damage in children with spastic unilateral cerebral palsy (uCP) is challenging, particularly in clinical settings. In this study, we developed and validated a deep learning-based pipeline to automatically quantify lesion-free brain volumes. Using T1-weighted and FLAIR MRI data from 35 patients (aged 5–15 years), we trained models to segment brain structures and lesions, utilizing an automatic label generation workflow. Validation was performed on 54 children with CP (aged 7–16 years) using quantitative and qualitative metrics, as well as an independent dataset of 36 children with congenital or acquired brain anatomy distortions (aged 1–17 years). Clinical evaluation examined the correlation of lesion-free volumes with visual-based assessments of lesion extent and motor and visual outcomes. The models achieved robust segmentation performance in brains with severe anatomical alterations and heterogeneous lesion appearances, identifying reduced volumes in the affected hemisphere, which correlated with lesion extent (*p* < 0.05). Further, regional lesion-free volumes, especially in subcortical structures such as the thalamus, were linked to motor and visual outcomes (*p* < 0.05). These results support the utility of automated lesion-free volume quantification for exploring brain structure-function relationships in uCP.

## 1 Introduction

Cerebral Palsy (CP), one of the most common childhood-onset motor disabilities, is caused by non-progressive damage to the developing brain, resulting in permanent movement and/or posture disorders (Cans, 2000). Structural magnetic resonance imaging (MRI) is crucial for identifying the timing, extent, and location of brain injuries in CP (Himmelmann et al., 2017). Hence, visual examination of structural MRI can aid in diagnosing CP, determining subtypes and comorbidities, and classifying clinical outcomes (Accardo et al., 2004; Franki et al., 2020; Bax et al., 2006). To standardize evaluation of MRI findings in CP, visual-based classification systems such as the MRI classification system (Himmelmann et al., 2017) and the semi-quantitative MRI (sqMRI) scale (Fiori et al., 2014) have been developed. The MRI classification system is a qualitative classification based on pathogenic patterns occurring at different stages of brain development, including maldevelopments, predominant white matter injury, predominant gray matter injury, miscellaneous findings, and normal findings (Himmelmann et al., 2017). The sqMRI scale is used to assess brain lesion locations and extent (Fiori et al., 2014). While these methods have high inter-rater reliability, they are limited by their reliance on time-consuming subjective evaluations (e.g., 15–30 min per patient for sqMRI).

Quantitative assessment methods, such as volumetric analysis, offer a more objective approach to brain assessment compared to visual MRI inspection alone. Traditional brain volumetric analysis tools, such as FreeSurfer (Fischl, 2012), icobrain v1.0–v5.9 (Struyfs et al., 2020), and childmetrix (Phan et al., 2021), rely on anatomically typical or nearly typical priors. Consequently, these methods often fail in the presence of structural pathology (i.e., large anatomical alterations) (Amorosino et al., 2020; Radwan et al., 2021). To address this limitation, methods specifically designed for large anatomical alterations have been developed. For example, a patch-based learning method has been applied to patients with conditions such as normal pressure hydrocephalus (Roy et al., 2015). Similarly, an atlas-based approach was specifically designed to automate brain lesion characterization in children with CP (Pagnozzi et al., 2016), highlighting the clinical significance of white matter (WM) and gray matter (GM) lesions in this population. Outperforming traditional approaches based on atlas, deep learning models provide an alternative for brain segmentation (Akkus et al., 2017). For example, the deep learning model icobrain-dl has shown improved measurement reproducibility in both pediatric and adult populations compared to atlas-based methods such as childmetrix and icobrain v5.9 (Simarro et al., 2024). However, the ability of deep learning models to generalize to unseen data remains a challenge, especially in patients with severe morphological abnormalities and heterogeneous lesion appearances (e.g., encephalomalacia caused by local infarct) (Simarro et al., 2023). This limitation could be addressed by incorporating training data representative of the target population, as demonstrated in prior research focused on patients with ventriculomegaly (Shao et al., 2019).

In this study, we aimed to develop and validate a deep learning-based pipeline tailored for quantitative brain measurements in children with spastic unilateral CP (uCP), characterized by motor impairments predominantly affecting one side of the body (Rosenbaum et al., 2007). We hypothesized that lower lesion-free volumes (i.e., volumes of unaffected brain structures) would be associated with greater brain lesion extent, as assessed using the sqMRI scale. Given that brain lesions in CP are non-progressive, we also explored the clinical relevance of lesion-free volumes to better understand structure-function relationships influenced by neuroplasticity. This involved examining differences in regional volumes between the affected and less-affected hemispheres and exploring structure-function associations related to motor and visual outcomes.

## 2 Materials and methodology

### 2.1 Datasets

In this study, data were obtained from two different cohorts of children with uCP. The first cohort was used to develop and validate the proposed model, with the data divided into a training and test set. Data from the second cohort were used to evaluate the model's performance on an independent dataset. For the clinical validation, we generated a merged dataset consisting of both the test and independent datasets. Demographic information and clinical characteristics of the datasets are presented in Table 1. The acquisition MRI protocols are provided in Supplementary material.

**Table 1:** Demographic information and clinical characteristics across training, test and independent CP datasets.

		**Training**	**Test**	**Independent**
Number of children		35	20	34
Female		19 (54.3%)	8 (40.0%)	15 (44.1%)
Right-sided uCP		14 (50.0%)^*a*^	8 (40.0%)	16 (47.1%)
Age range in years		9.0 ± 2.7	12.0 ± 1.9	11.5 ± 2.8
		(5.0–15.0)	(7.0–15.0)	(7.1–16.0)
Total sqMRI		10.0 ± 4.5^*a*^	11.0 ± 5.7	11.0 ± 5.9
		(0.0–16.5)^*a*^	(0.5–21.5)	(0.0–26.0)
AHA		59.0 ± 12.0^*a*^	68.0 ± 17.1^*b*^	76.0 ± 15.5
		(43–89)^*a*^	(34–89)^*b*^	(46–100)
MACS	I	7 (25.0%)^*a*^	8 (44.4%)^*b*^	20 (58.8%)
	II	10 (35.7%)^*a*^	6 (33.3%)^*b*^	10 (29.4%)
	III	11 (39.3%)^*a*^	4 (22.2%)^*b*^	4 (11.8%)
MRICS	Maldevelopments	0 (0.0%)	0 (0.0%)	2 (5.9%)
	Predominant WM injury	18 (51.4%)	10 (50.0%)	23 (67.6%)
	Predominant GM injury	16 (45.7%)	9 (45.0%)	6 (17.6%)
	Miscellaneous	0 (0.0%)	0 (0.0%)	1 (2.9%)
	Normal	0 (0.0%)	1 (5.0%)	2 (5.9%)

Mean ± standard deviation (min–max).

uCP, unilateral cerebral palsy; sqMRI, semi-quantitative MRI; AHA, assisting hand assessment; MACS, manual ability classification system; MRICS, MRI classification system; WM, white matter; GM, gray matter.

^*a*^Data available for 28 children.

^*b*^Data available for 18 children.

For the datasets included in the current study, written parental informed consent was obtained for all children, according to the Declaration of Helsinki. Additionally, children aged 12 years or above were asked for their written assent. Both studies were approved by the local Ethical Committee of UZ Leuven, Belgium (S67752 and S62906).

#### 2.1.1 Training and test CP datasets

Fifty-five children with uCP, aged between 5 and 15 years, were recruited between May 2014 and April 2017 as part of a previous project (FWO project G087213N). Brain lesions (FLAIR hyperintensities and cavities) were manually annotated by a neuroradiologist (A.M.R.) using ITK-snap (Yushkevich et al., 2019). Thirty-five children were randomly assigned to the training dataset to optimize the weights of the deep learning models. The remaining 20 patients formed the test set and were exclusively used to evaluate the model's performance, without being involved in any training steps.

#### 2.1.2 Independent CP dataset

The independent dataset consisted of 34 children diagnosed with uCP, aged between 7 to 16 years. The data was collected as part of a previous project performed between 2021 and 2023 (FWO project G0C4919N), where motor and visual performance was assessed. A detailed description of the motor characteristics is presented in Table 1, while the visual assessments are provided in Supplementary Table S1.

#### 2.1.3 Distorted brain benchmark dataset

The independent distorted brain benchmark dataset consists of 36 children with congenital or acquired brain anatomy distortions, aged between 1 to 17 years, selected from the EMEDEA-PED archive (Amorosino et al., 2022). One patient from the original dataset was excluded due to an age below 1 year. The distorted brains involve alterations in the normal shape of cerebral structures (e.g., cortex, ventricles), the absence of entire structures (e.g., corpus callosum, vermis), disrupted spatial relationships among structures, and volume changes in normal tissues or anatomical regions, including agenesis of the corpus callosum, posterior fossa malformations, malformations of cortical development, and severe brain distortions related to complex malformations or lesions (Amorosino et al., 2022).

We selected this fully independent pediatric dataset of T1-weighted images to validate the model's performance in patients with brain anatomy distortions. Ground truth annotations were generated using a semi-automatic procedure guided by an expert; for further details, we refer to Amorosino et al. (2022).

### 2.2 Clinical and radiological assessment

#### 2.2.1 Semi-quantitative MRI scores

MR images from the training, test, and independent datasets were classified using the sqMRI scale. Brain lesions were mapped onto a six-axial slice template, and brain damage was assessed across various regions, with higher scores indicating greater lesion extent. Lesion extent in the frontal, parietal, temporal, and occipital lobes was scored from 0 to 3, as per sqMRI scoring, with each hemisphere evaluated separately. Additionally, brain structures, including the lenticular nucleus (sum of globus pallidus and putamen), caudate nucleus, posterior limb of the internal capsule (PLIC), thalamus, and cerebellum, were scored on a binary scale (i.e., presence or absence of lesions). For a detailed description of the sqMRI methodology, see Fiori et al. (2014).

#### 2.2.2 Clinical tests for motor ability

Manual ability was classified using the Manual Ability Classification System (MACS) (Eliasson et al., 2006), and bimanual performance was assessed via the Assisting Hand Assessment (AHA) (Holmefur and Krumlinde-Sundholm, 2016). MACS assesses the ability of children with CP to handle objects in everyday activities, where higher levels indicate greater impairment. The AHA evaluates the spontaneous use of the hand with greater impairment during bimanual activities through a video-recorded semi-structured play session. For the AHA, a lower scores indicates greater impairment.

#### 2.2.3 Clinical tests for vision

Clinical tests for vision included the assessment of visual functions (i.e, the performance of components of the visual system) and functional vision (i.e., visual task-related ability) (Bennett et al., 2019). Visual functions were assessed through several tests; (1) binocular stereoacuity was evaluated using the fly and circle subtests of the Titmus Stereo Fly Test (2018)^1^; (2) binocular visual acuity was measured with the Freiburg Visual Acuity Test (Bach, 1996); (3) motor-free visual-perceptual skills were assessed using five subtests of the Test of Visual Perceptual Skills, Fourth Edition (TVPS-4), including visual discrimination, spatial relationships, form constancy, visual figure-ground, and visual closure (Martin, 2017); and (4) motor-dependent visual-perceptual skills were assessed using the subtest of the Beery Buktenica Test of Visual-Motor Integration, Sixth Edition (Beery-VMI) (Beery, 1989). Lower scores in each of these tests indicated greater impairment, except for the Freiburg Visual Acuity Test, where higher scores reflected greater impairment. To assess functional vision, the Flemish Cerebral Visual Impairment Questionnaire (FCVIQ) (Ortibus et al., 2011), a 46-item binary-response screening tool completed by caregivers, was implemented. The total FCVIQ score was calculated by summing the positive answers, with higher scores indicating greater impairment.

For a detailed description of the clinical tests for motor ability, vision and relative scoring system, see Crotti et al. (2024).

### 2.3 Proposed MRI quantification pipeline

**Figure 1:**
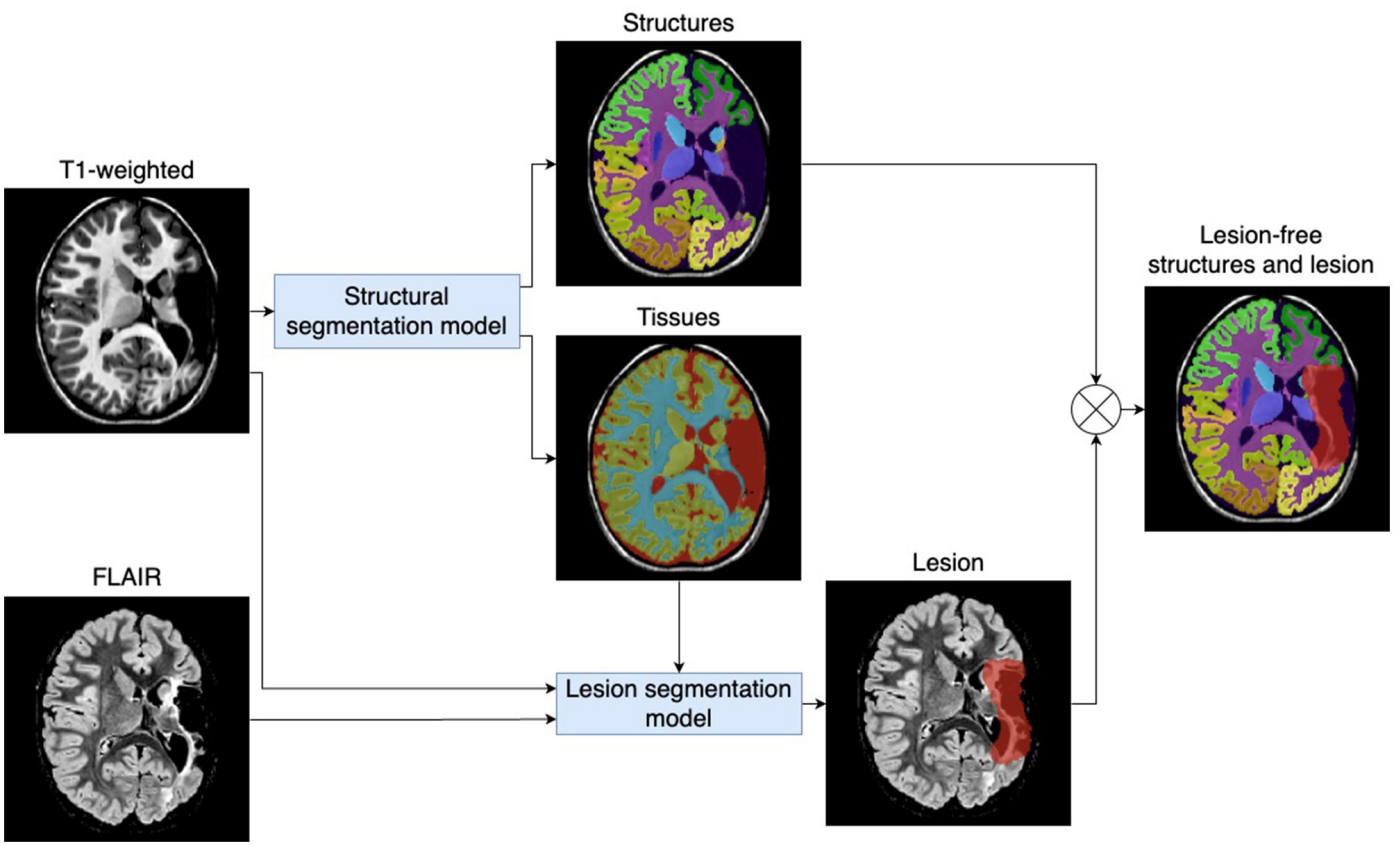
Proposed pipeline for automatic quantification of brain structures and lesions. The pipeline includes two deep learning models that process 3D T1-weighted and 3D FLAIR images to automatically quantify brain structures and lesions (e.g., periventricular leukomalacia and cavities).

The proposed pipeline segments and quantifies brain structures and lesions using 3D T1-weighted and 3D FLAIR images. Two deep learning models were trained for this purpose: one for structural segmentation and another, building on the first, for lesion segmentation. Figure 1 outlines the proposed pipeline.

#### 2.3.1 Structural segmentation model

The structural segmentation model processes T1-weighted images to segment brain tissue, including WM, GM, and cerebrospinal fluid (CSF), as well as 22 anatomical brain structures (see a detailed list in Supplementary material). The structural segmentation model was based on the extensively validated deep learning model of icobrain-dl, which accurately quantifies brain structures in both pediatric and adult populations where there are no severe anatomical alterations of the brain (Simarro et al., 2024). We retrained the model by fine-tuning its pre-existing weights using the training dataset, which consisted of patients with severe lesions and anatomical alterations. Training labels were generated as detailed in Section 2.5.

#### 2.3.2 Lesion segmentation model

The lesion segmentation model processes T1-weighted and FLAIR images, as well as tissue segmentation generated by the structural segmentation model described in Section 2.3.1 (i.e., WM, GM, CSF, or background of the image). The model automatically segments FLAIR hyperintensities (e.g. periventricular leukomalacia lesions) and T1-weighted/FLAIR hypointensities (e.g. cavities). The model initialized its weights using He weight initialization (He et al., 2015), and was optimized using manual brain lesion annotations of the training set (see Section 2.1.1).

#### 2.3.3 Lesion-free volume

Based on the segmentation of brain structures and lesions generated by the deep learning models, we calculated the lesion-free volume, representing the volume of brain structures unaffected by lesions (see Equation 1).


(1) $$\begin{array}{l} Lesion\text{-}free\;volume=Total\;volume-Lesion\;volume \end{array}$$

### 2.4 Model architecture, preprocessing and postprocessing

Both models utilized established 3D U-net deep learning architecture (Çiçek et al., 2016). Training continued until the validation loss converged, with validation conducted on a subset of five randomly selected patients from the training dataset. Models utilized a loss function combining soft Dice loss and weighted categorical cross-entropy. TensorFlow 2.6 was used for implementation. Preprocessing steps included affine registration to MNI space using NiftyReg (Ourselin et al., 2001) and intensity normalization to minimize scanner variability. First, intensities were clipped at the 1st and 99th percentiles to mitigate the impact of outliers. Then, normalization was performed using a variation of *z*-scoring, where the function was computed for values above the 10th percentile, prioritizing the median over the mean. The standard deviation was calculated within the 90th percentile range. Postprocessing included segmentation of WM lobes and PLIC by integrating the WM segmentation from the deep learning model, with localization information from atlases (Mori et al., 2005). For a detailed description of the preprocessing and implementation methods, we refer to Simarro et al. (2024).

### 2.5 Training labels generations

**Figure 2:**
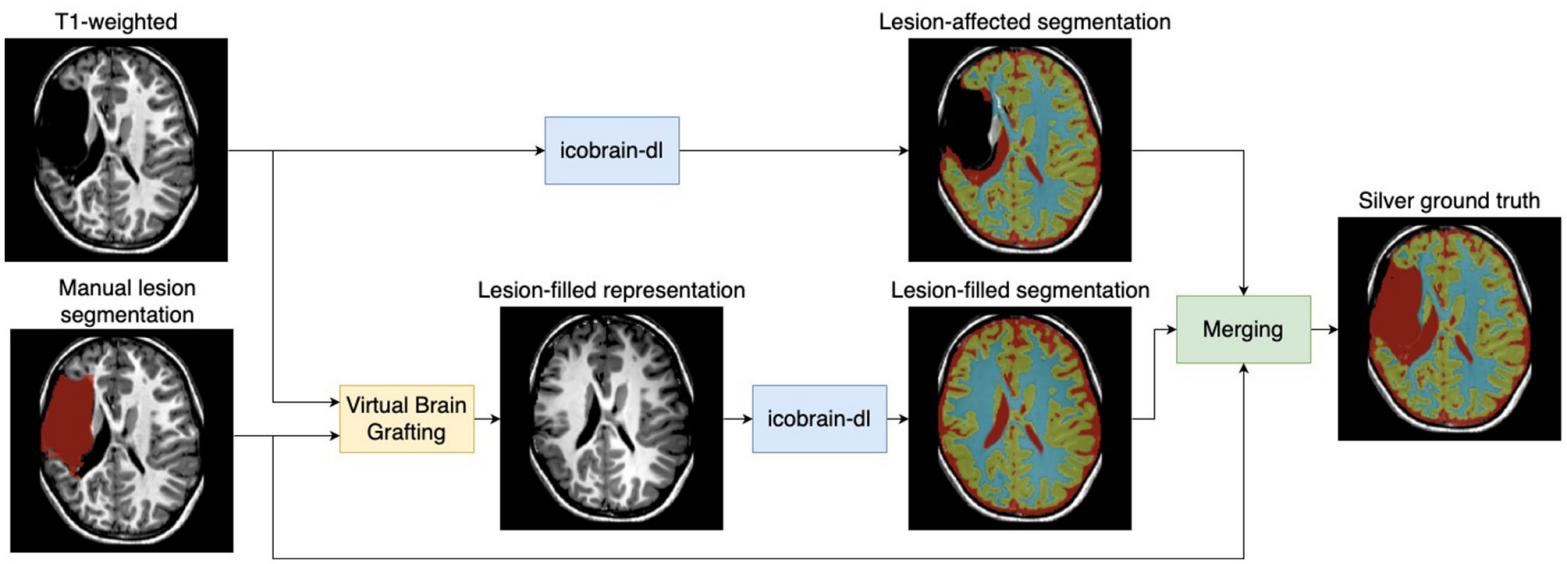
Workflow for generating silver ground truth labels. The methodology utilized icobrain-dl, a pre-trained deep learning model, on T1-weighted images to predict a *lesion-affected segmentation* (noted for lacking accuracy). Manual lesion segmentation was combined with the T1-weighted image using Virtual Brain Grafting (Radwan et al., 2021) to generate a *lesion-filled representation*, subsequently segmented by icobrain-dl to produce *lesion-free segmentation*. The *silver ground truth* was created by merging both segmentations.

Manual annotation of tissue and structures is highly time-consuming and not feasible for large datasets. Therefore, we proposed an efficient methodology to obtain labels, also referred to as *silver ground truth* (see Figure 2). Starting with labels predicted by icobrain-dl, we obtained the *lesion-affected segmentation*, which lacked accurate labeling in the lesion areas. In parallel, we combined the manual lesion segmentation labels with the T1-weighted image to generate a *lesion-filled representation* using Virtual Brain Grafting (Radwan et al., 2021). The result was then segmented using icobrain-dl to generate the *lesion-filled segmentation*, and both segmentations were merged to create the *silver ground truth* according to the following conditions, which were empirically defined on the training set:

- Outside the dilated manual lesion mask, the lesioned segmentation was used.
- Inside the dilated manual lesion mask, and when the intensity of the T1-weighted image fell within the 1st and 95th percentiles of the CSF intensity in the lesioned segmentation, the silver ground truth was labeled as CSF.
- Inside the dilated manual lesion mask and outside the T1-weighted intensity range of CSF, the lesion-filled segmentation was used.

### 2.6 Statistical analysis

The technical performance of the algorithm was evaluated both quantitatively and qualitatively. In the clinical performance, we tested the hypothesis that lower lesion-free volumes correlate with greater brain lesion extent, as assessed using the sqMRI scale. Additionally, we investigated the differences in lesion-free regional volumes between affected and less-affected hemispheres, and explored the correlations between the volumes of the affected hemisphere and motor and visual scores. In contrast to disjunction testing, where all tests are evaluated together and rejecting the joint null hypothesis requires at least one significant result (necessitating alpha adjustment), we explored brain structure-function relations through individual testing. In this approach, each structure-function relation is evaluated independently, and significance must be achieved individually to reject its corresponding null hypothesis. Consequently, no nominal alpha level adjustment (i.e., correction for multiple comparisons) was applied (Rubin, 2021). Significance levels were assessed at *p*-value 0.05, 0.01, and 0.001.

#### 2.6.1 Technical validation

##### 2.6.1.1 Quantitative validation

Quantitative validation involved comparing the overlap between the structures predicted by the model and the silver ground truth in the test dataset as well as the manual annotations in the distorted brain benchmark dataset. To align with the annotations in the distorted brain benchmark dataset, regions were classified as cerebrospinal fluid, GM, WM, deep GM (including thalamus, putamen, caudate and pallidum), brainstem (including midbrain, pons and medulla), and cerebellum. Segmentation accuracy was assessed using the Dice similarity coefficient, and the 95th percentile of the Hausdorff distance. The Dice similarity coefficient quantifies the overlap between segmentation masks, ranging from 0 for no overlap to 1 for perfect agreement, while the Hausdorff distance measures maximal contour distance (in millimeters) between masks, with smaller values indicating greater similarity.

##### 2.6.1.2 Qualitative validation

Qualitative validation was based on visual assessments of segmentation quality by two independent raters in the independent dataset. Prior to rater evaluation, and inspired by the qualitative evaluation proposed in Radwan et al. (2021), we defined a protocol for visual quality assessment.

The protocol aimed to measure the quality of the segmentation and its impact on final volume measurements. The evaluation process involved a neuroradiologist (A.M.R.) and a child neurologist (E.O.), who visually assessed the segmentations on high-resolution multi-frame panels of axial and coronal slices from both T1-weighted and FLAIR images. Parcellation defects were defined as unlabeled or erroneously labeled voxels within a region. Those were considered minor if not exceeding ~10% of a region's volume, intermediate if between 10%–25%, and major if exceeding 25% of a region's volume as estimated by the experts. The quality of the segmentation was evaluated across several regions, including WM lobes, cortical GM lobes, and subcortical structures, using the following categories:

- Rejected: segmentation fails to capture anatomy, with more than three intermediate defects or one major defect.
- Approved with remarks: segmentation captures overall volume but has up to three intermediate-scale defects or more than three minor defects.
- Approved: accurate segmentation with up to three minor defects.

Inter-rater reliability was assessed by computing the average random raters' intraclass correlation coefficient. Moderate reliability was defined by an intraclass correlation coefficient between 0.5 and 0.75, while good reliability was defined between 0.75 and 0.9 (Koo and Li, 2016).

#### 2.6.2 Clinical validation

##### 2.6.2.1 Correlation analysis with sqMRI scores

Spearman's rank correlation was used to analyze the relationship between lesion-free volumes and sqMRI scores of the WM in the different lobes (scored from 0 to 3). For subcortical structures, significant differences in regional volumes between the regions with and without sqMRI findings were assessed using the non-parametric Mann-Whitney U test. Confidence intervals with a confidence level of 95% were calculated using bootstrapping.

#### 2.6.3 Exploratory analysis using the lesion-free volume

##### 2.6.3.1 Relative difference between regional volumes in affected and less-affected hemispheres

The affected brain hemisphere in uCP is defined as the one contralateral to the clinical side (i.e. the side with predominant motor impairments). We computed the lesion-free regional volume differences between hemispheres, and tested the significance of these differences using the non-parametric Wilcoxon signed-rank test.

##### 2.6.3.2 Exploratory analysis with clinical tests for motor ability and vision

We investigated the Spearman's rank correlations between lesion-free volumes in the affected hemisphere and motor and visual scores.

## 3 Results

### 3.1 Quantitative validation of the segmentation model

In the independent CP dataset, the brain structures segmented by the model achieved a Dice similarity coefficient greater than 0.95 and a 95th percentile Hausdorff distance below 1.2 mm across all brain structures compared to the silver ground truth in 20 patients from the test set. See Supplementary Table S2 for detailed information on the quantitative validation of the brain regions.

**Figure 3:**
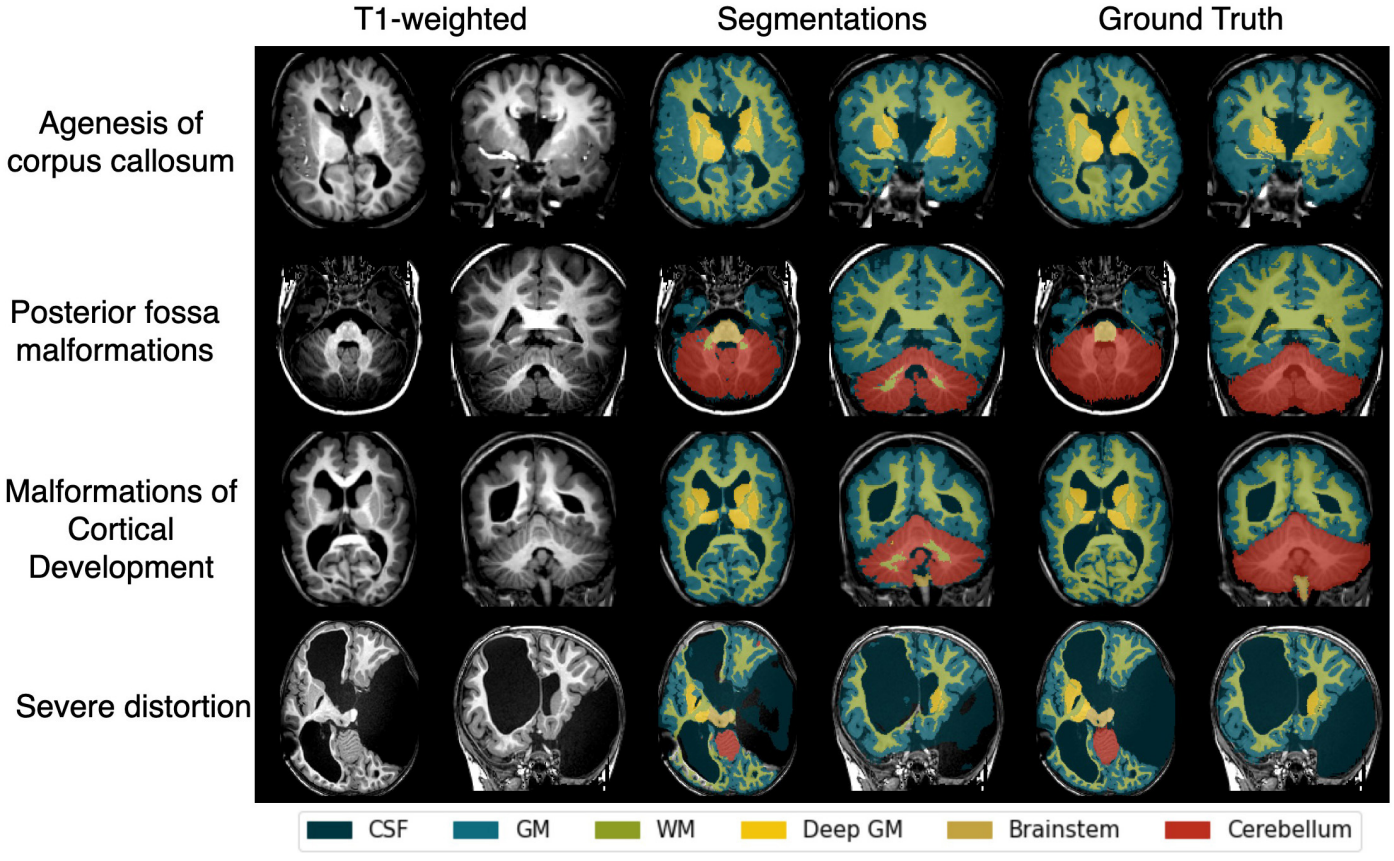
Examples of automatic segmentation compared with manual annotations from the distorted brain benchmark dataset, including patients with congenital or acquired brain anatomy distortions such as agenesis of the corpus callosum, posterior fossa malformations, malformations of cortical development, and severe brain distortions related to complex malformations or lesions. CSF, cerebrospinal fluid; WM, white matter; GM, gray matter.

In the distorted brain benchmark dataset, as shown in Table 2, the model achieved a Dice similarity coefficient greater than 0.74 and a 95th percentile Hausdorff distance below 11.2 mm across all parenchymal regions (i.e., excluding CSF) compared to manual annotations. Figure 3 presents the automatic segmentation compared with manual annotations, showcasing the performance of the model in cases with congenital or acquired brain anatomy distortions including agenesis of the corpus callosum, posterior fossa malformations, malformations of cortical development, and severe brain distortions.

**Table 2:** Segmentation overlap in patients with congenital or acquired brain anatomy distortions.

	**Dice similarity coefficient**	**Hausdorff distance 95th percentile**

CSF	0.67 (0.1)	5.65 (3.25)
GM	0.8 (0.06)	2.67 (2.59)
WM	0.83 (0.05)	1.96 (0.83)
Deep GM	0.74 (0.14)	5.74 (5.99)
Brainstem	0.84 (0.04)	7.15 (13.89)
Cerebellum	0.8 (0.11)	11.19 (12.13)

Mean (standard deviation) of the dice similarity coefficient and Haus-dorff distance 95th percentile of the segmentation produces by the model and the manual annotation in the distorted brain benchmark dataset. CSF, cerebrospinal fluid; WM, white matter; GM, gray matter.

### 3.2 Qualitative validation of the segmentation models

**Figure 4:**
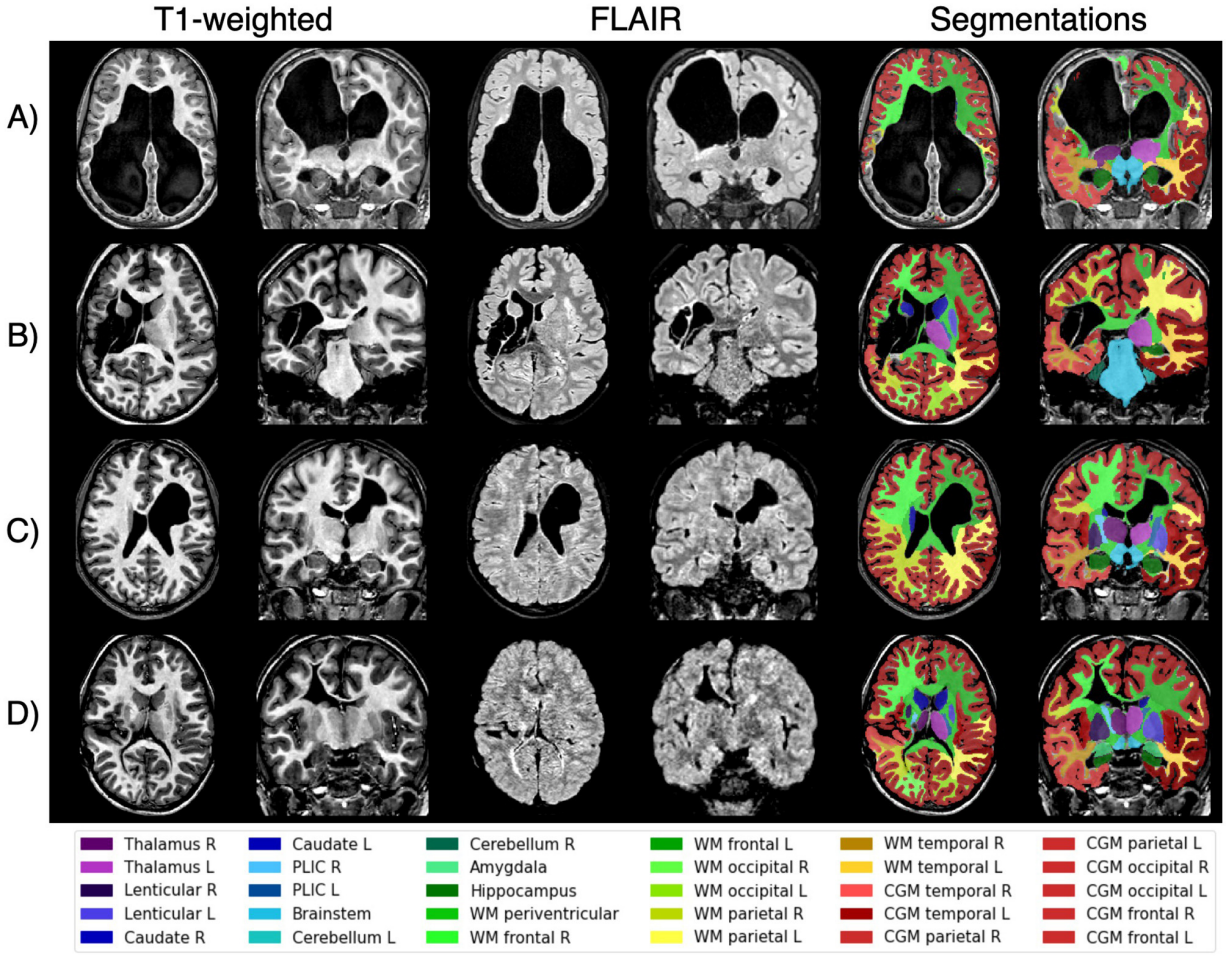
Examples of patients with unilateral cerebral palsy from the independent set illustrate varying segmentation quality as evaluated by the raters. Patient **(A)** was the only case where both raters rejected the white matter and cortical gray matter segmentations, likely due to extreme lesion appearance and limited healthy tissue. In contrast, the segmentations for patients **(B–D)** were approved despite their severe and heterogeneous lesions, demonstrating the model's robustness in handling diverse cases in children with unilateral cerebral palsy. L, left; R, right; PLIC, posterior limb of the internal capsule; WM, white matter; CGM, cortical gray matter.

Table 3 presents the segmentation quality as evaluated by each rater for various brain regions in the independent dataset. The intraclass correlation coefficient between raters was 0.75, indicating moderate/good agreement. Subcortical structure segmentations were approved (with or without remarks) for every patient. For the 34 patients in the dataset, between 1 and 8 segmentations were rejected for WM, and between 2 and 5 for CGM. Figure 4 shows the automatic segmentation of patients with varying quality, ranging from the only patient whose WM and CGM segmentations were rejected by both raters (Figure 4A) to patients with approved segmentations (Figures 4B–D) despite anatomical alteration and heterogeneous lesion appearance.

**Table 3:** Visual evaluation of segmentation quality by each of the expert raters in the independent datasets.

	**Structures**		**White matter**		**Cortical gray matter**	
	**R1**	**R2**	**R1**	**R2**	**R1**	**R2**
Rejected	0 (0.0%)	0 (0.0%)	8 (23.5%)	1 (2.9%)	2 (5.9%)	5 (14.7%)
Approved with remarks	9 (26.5%)	9 (26.5%)	17 (50.0%)	13 (38.2%)	14 (41.2%)	10 (29.4%)
Approved	25 (73.5%)	25 (73.5%)	9 (26.5%)	20 (58.8%)	18 (52.9%)	19 (55.9%)

The intraclass correlation coefficient between raters is 0.75. R1, rater 1; R2, rater 2.

### 3.3 Clinical validation

#### 3.3.1 Correlation with sqMRI scales

Table 4 presents the correlations between the regional lesion-free volume and the regional sqMRI across the test, independent, and merged CP datasets. In the merged dataset, significant correlations were found between lesion-free volume and sqMRI scores in the frontal, parietal, and temporal lobes (|*r*| = 0.58–0.77, *p* < 0.001), as well as in the left occipital lobe (|*r*| = 0.35, *p* < 0.01). For brain structures with binary sqMRI scores, significant volume differences were identified between regions with and without sqMRI findings in the thalamus, caudate nucleus, and lenticular nucleus (*U* = 0 − 109, *p* < 0.05). The cerebellum was excluded due to the absence of sqMRI-assessed lesions.

**Table 4:** Lesion-free volumes were consistent with the semi-quantitative lesion burden scores (sqMRI).

	**Test (*n* = 20)**	**Independent (*n* = 34)**	**Merged (*n* = 54)**
**Spearman correlation (** *r* **)**			
WM frontal L	–0.77 (–0.4, –0.91)^***^	–0.74 (–0.47, –0.87)^***^	–0.75 (–0.58, –0.85)^***^
WM frontal R	–0.81 (–0.52, –0.94)^***^	–0.75 (–0.5, –0.87)^***^	–0.77 (–0.61, –0.87)^***^
WM occipital L	–0.57 (–0.15, –0.8)^**^	–0.24 (0.17, –0.58)	–0.35 (–0.06, –0.59)^**^
WM occipital R	–0.12 (–0.57, 0.42)	0.05 (–0.34, 0.4)	0.0 (–0.3, 0.29)
WM parietal L	–0.85 (–0.74, –0.93)^***^	–0.7 (–0.43, –0.85)^***^	–0.74 (–0.56, –0.84)^***^
WM parietal R	–0.74 (–0.42, –0.9)^***^	–0.81 (–0.71, –0.9)^***^	–0.77 (–0.63, –0.87)^***^
WM temporal L	–0.74 (–0.39, –0.87)^***^	–0.45 (–0.07, –0.72)^**^	–0.58 (–0.31, –0.75)^***^
WM temporal R	–0.7 (–0.31, –0.87)^***^	–0.5 (–0.14, –0.75)^**^	–0.59 (–0.35, –0.75)^***^
**Mann–Whitney** *U***-test (***U***)**			
Thalamus L	17 (2, 42)^*^	18 (0, 60)^*^	78 (13, 205)^**^
Thalamus R	15 (1, 40)^*^	43 (12, 93)^*^	109 (48, 202)^***^
Caudate L	0^**^	28 (8, 46)	42 (8, 119)^**^
Caudate R	9 (0, 18)	0^*a*^	17 (0, 51)^*^
Lenticular L	0^**^	0^*a*^	0^***^
Lenticular R	0^***^	4 ^*a*^	11 (2, 36)^***^
PLIC L	17 (5, 41)^*^	78 (40, 103)	163 (89, 257)^*^
PLIC R	27(9, 47)	125(109, 132)	257 (156, 335)

An inverse Spearman correlation was found between lesion-free volumes and sqMRI scores in WM lobes (sqMRI: 0–3). For subcortical structures, significant differences in regional volumes between the regions with and without sqMRI findings were found using the non-parametric Mann–Whitney *U*-test. The analysis was conducted on the test, independent, and merged CP datasets. For WM lobes: Spearman correlation coefficient with 95% confidence intervals. For subcortical structures: Mann–Whitney *U* with 95% confidence intervals.

L, left; R, right; WM, white matter; CGM, cortical gray matter; PLIC, posterior limb of the internal capsule.

^*a*^Confidence intervals cannot be computed due to the presence of only one positive sample.

^*^p < 0.05.

^**^p < 0.01.

^***^p < 0.001.

### 3.4 Exploratory analysis

#### 3.4.1 Regional volume differences between affected and less-affected hemispheres

Table 5 shows the relative regional volume differences between hemispheres (affected minus less-affected). In the merged dataset, all the cerebral regions showed lower volumes in the affected hemisphere, with relative reductions ranging from 5% (occipital CGM) to 34% (PLIC). Significant differences were observed in all these regions, including the thalamus, caudate, lenticular nucleus, PLIC, occipital WM, WM and CGM in the parietal, frontal, and temporal lobes (*p* < 0.001), as well as occipital CGM (*p* < 0.05). In contrast, the cerebellum showed a trend toward greater volume in the affected hemisphere, though this difference was not statistically significant.

**Table 5:** Relative difference between regional volumes in affected and less-affected hemispheres.

		**Training (*n* = 35)**	**Test (*n* = 20)**	**Independent (*n* = 34)**	**Merged (*n* = 54)**
WM	Parietal	–30.5 (30.1)^***^	–35.4 (40.3)^***^	–20.8 (30.0)^***^	–25.9 (34.6)^***^
		Occipital	–17.3 (20.8)^***^	–19.6 (30.4)^*^	–12.6 (23.7)^**^	–15.0 (26.4)^***^
		Frontal	–22.0 (20.4)^***^	–39.8 (48.0)^***^	–11.4 (15.7)^***^	–21.2 (33.8)^***^
		Temporal	–14.5 (22.4)^***^	–23.1 (38.6)^**^	–9.1 (17.6)^**^	–13.9 (27.6)^***^
		Total	–22.0 (20.5)^***^	–31.6 (35.8)^***^	–12.5 (15.8)^***^	–19.1 (26.3)^***^
CGM	Parietal	–19.6 (27.2)^***^	–26.7 (35.3)^***^	–17.7 (27.5)^***^	–20.8 (30.7)^***^
		Occipital	–7.6 (11.0)^***^	–8.1 (14.8)^*^	–4.3 (12.4)	–5.6 (13.4)^*^
		Frontal	–12.8 (12.6)^***^	–27.1 (38.7)^***^	–7.3 (10.4)^***^	–14.1 (26.0)^***^
		Temporal	–12.2 (20.0)^***^	–23.4 (35.2)^***^	–6.1 (12.6)^*^	–12.1 (24.5)^***^
		Total	–13.5 (15.9)^***^	–22.4 (28.5)^***^	–8.5 (11.6)^***^	–13.3 (20.3)^***^
Structures	Thalamus	–36.0 (28.0)^***^	–48.2 (45.8)^***^	–18.3 (26.4)^***^	–28.7 (37.2)^***^
		Caudate	–35.6 (42.1)^***^	–56.4 (63.5)^***^	–11.7 (18.5)^***^	–27.2 (45.5)^***^
		Lenticular	–31.2 (42.1)^***^	–49.7 (62.5)^***^	–9.4 (22.3)^**^	–23.4 (45.2)^***^
		PLIC	–67.2 (60.7)^***^	–69.9 (65.9)^***^	–16.0 (53.1)^**^	–34.7 (63.3)^***^
		Cerebellum	2.4 (3.2)^***^	1.8 (4.2)	1.5 (7.5)	1.6 (6.6)

Mean (standard deviation) of affected minus less-affected, divided by the mean of both. Significant differences were assessed using the non-parametric Wilcoxon signed-rank test. Volumes from the training dataset were derived from the silver ground truth, while volumes from the test, independent, and merged CP datasets were computed using the proposed pipeline.

WM, white matter; CGM, cortical gray matter; PLIC, posterior limb of the internal capsule.

^*^p < 0.05.

^**^p < 0.01.

^***^p < 0.001.

#### 3.4.2 Correlation with clinical tests for motor ability

As shown in Table 6, motor ability (AHA and MACS) correlated significantly with the regional lesion-free volumes of the affected hemisphere in the thalamus, caudate, and lenticular nucleus, as well as WM and CGM in parietal, frontal, and temporal lobes (|*r*| = 0.4–0.55, *p* < 0.01) in the merged dataset. Significant correlations were found between AHA and occipital WM and PLIC (|*r*| = 0.28–0.32, *p* < 0.05), while no significant correlations were observed between the motor ability test and the occipital CGM lobe or the cerebellum.

**Table 6:** Correlations between lesion-free volume in the affected hemisphere and motor scores.

	**Parietal**	**Occipital**	**Frontal**	**Temporal**	**Total**
**WM**					
AHA	0.46 (0.21, 0.66)^***^	0.32 (0.05, 0.54)^*^	0.49 (0.24, 0.69)^***^	0.45 (0.2, 0.66)^***^	0.53 (0.29, 0.72)^***^
MACS	–0.4 (–0.11, –0.61)^**^	–0.27 (–0.53, 0.03)	–0.5 (–0.24, –0.68)^***^	–0.44 (–0.13, –0.66)^**^	–0.48 (–0.21, –0.68)^***^
**CGM**					
AHA	0.55 (0.32, 0.72)^***^	0.1 (–0.21, 0.38)	0.42 (0.14, 0.65)^**^	0.43 (0.14, 0.65)^**^	0.51 (0.26, 0.7)^***^
MACS	–0.52 (–0.25, –0.72)^***^	–0.09 (–0.39, 0.22)	–0.43 (–0.14, –0.65)^**^	–0.41 (–0.09, –0.65)^**^	–0.48 (–0.21, –0.69) ^***^
	**Thalamus**	**Caudate**	**Lenticular**	**PLIC**	**Cerebellum**
**Structures**					
AHA	0.54 (0.27, 0.73)^***^	0.41 (0.13, 0.62)^**^	0.45 (0.19, 0.66)^***^	0.28 (–0.01, 0.54)^*^	–0.25 (–0.51, 0.05)
MACS	–0.52 (–0.24, –0.71)^***^	–0.44 (–0.18, –0.66)^**^	–0.45 (–0.19, –0.65)^***^	–0.25 (–0.52, 0.05)	0.19 (–0.12, 0.45)

Significant Spearman correlations between lesion-free volume in the affected hemisphere and motor scores, MACS and AHA were observed in most of the regions in the merged dataset (*n* = 54). Spearman correlation coefficient and 95% confidence intervals.

WM, white matter; CGM, cortical gray matter; PLIC, posterior limb of the internal capsule; AHA, assisting hand assessment; MACS, manual ability classification system.

^*^p < 0.05.

^**^p < 0.01.

^***^p < 0.001.

#### 3.4.3 Correlation with clinical tests for vision

Table 7 shows the correlation between the lesion-free volume of the affected hemisphere and visual tests. Titmus Stereo Fly, Freiburg Visual Acuity, TVPS-4, and FCVIQ showed significant correlations with total WM volume (|*r*| = 0.35–0.51, *p* < 0.05). The total score of the functional vision questionnaire, FCVIQ, correlated with each WM lobe independently (|*r*| = 0.35–0.49, *p* < 0.05). Notably, the frontal and temporal lobes correlated with all the motor-free visual-perceptual skills measured by the TVPS-4 scores (|*r*| = 0.35–0.43, *p* < 0.05). The thalamus also exhibited significant correlations with Titmus Stereo Fly, Freiburg Visual Acuity, TVPS-4 (excluding the spatial relationships subtest), Beery-VMI, and FCVIQ (|*r*| = 0.35–0.58, *p* < 0.05).

**Table 7:** Correlations between lesion-free volume in the affected hemisphere and clinical tests for vision scores.

	**Parietal**	**Occipital**	**Frontal**	**Temporal**	**Total**
**WM**					
Titmus Stereo Fly	0.32 (–0.02, 0.6)	0.28 (–0.1, 0.59)	0.36 (0.03, 0.62)^*^	0.27 (–0.13, 0.59)	0.4 (0.07, 0.66)^*^
Freiburg Visual Acuity	–0.39 (–0.02, –0.66)^*^	–0.28 (–0.58, 0.09)	–0.28 (–0.57, 0.09)	–0.27 (–0.57, 0.1)	–0.38 (–0.64, 0.0)^*^
TVPS-4 visual discrimination	0.34 (0.01, 0.59)	0.13 (–0.25, 0.47)	0.37 (0.08, 0.62)^*^	0.41 (0.01, 0.68)^*^	0.35 (0.0, 0.6)^*^
TVPS-4 spatial relationships	0.33 (–0.01, 0.62)	0.12 (–0.27, 0.48)	0.4 (0.09, 0.65)^*^	0.42 (0.05, 0.67)^*^	0.38 (0.05, 0.65)^*^
TVPS-4 form constancy	0.37 (0.02, 0.63) ^*^	0.24 (–0.11, 0.53)	0.41 (0.09, 0.66)^*^	0.35 (–0.03, 0.64)^*^	0.43 (0.08, 0.67)^*^
TVPS-4 visual figure–ground	0.28 (–0.03, 0.55)	0.18 (–0.15, 0.49)	0.35 (0.06, 0.59)^*^	0.39 (0.03, 0.64)^*^	0.36 (0.05, 0.6) ^*^
TVPS-4 visual closure	0.31 (–0.02, 0.56)	0.14 (–0.24, 0.46)	0.36 (0.06, 0.61)^*^	0.43 (0.07, 0.66)^*^	0.4 (0.09, 0.63)^*^
Beery-VMI	0.19 (–0.19, 0.52)	0.26 (–0.12, 0.56)	0.39 (0.04, 0.65)^*^	0.11 (–0.28, 0.47)	0.27 (–0.1, 0.58)
FCVIQ	–0.45 (–0.11, –0.69)^**^	–0.47 (–0.17, –0.7)^**^	–0.49 (–0.2, –0.66)^**^	–0.35 (–0.01, –0.61)^*^	–0.51 (–0.19, –0.71)^**^
**CGM**					
Titmus Stereo Fly	0.12 (–0.27, 0.49)	0.17 (–0.22, 0.52)	0.09 (–0.32, 0.45)	–0.07 (–0.46, 0.37)	0.11 (–0.3, 0.5)
Freiburg Visual Acuity	–0.11 (–0.47, 0.26)	0.04 (–0.33, 0.41)	–0.05 (–0.4, 0.32)	0.16 (–0.22, 0.51)	0.0 (–0.37, 0.37)
TVPS-4 visual discrimination	0.31 (–0.04, 0.58)	0.02 (–0.36, 0.42)	0.24 (–0.12, 0.54)	0.18 (–0.23, 0.53)	0.25 (–0.13, 0.56)
TVPS-4 spatial relationships	0.4 (0.01, 0.67) ^*^	0.17 (–0.22, 0.5)	0.34 (–0.04, 0.62)^*^	0.15 (–0.24, 0.5)	0.35 (–0.07, 0.64)^*^
TVPS-4 form constancy	0.18 (–0.19, 0.5)	0.03 (–0.35, 0.4)	0.16 (–0.22, 0.49)	–0.03 (–0.42, 0.39)	0.11 (–0.28, 0.47)
TVPS-4 visual figure–ground	0.22 (–0.12, 0.54)	–0.08 (–0.42, 0.28)	0.11 (–0.27, 0.45)	0.06 (–0.35, 0.43)	0.13 (–0.27, 0.48)
TVPS-4 visual closure	0.24 (–0.07, 0.51)	–0.07 (–0.41, 0.27)	0.17 (–0.2, 0.47)	–0.01 (–0.39, 0.38)	0.15 (–0.2, 0.46)
Beery-VMI	0.3 (–0.01, 0.57)	0.11 (–0.28, 0.44)	0.28 (–0.14, 0.6)	0.06 (–0.34, 0.41)	0.29 (–0.11, 0.59)
FCVIQ	–0.42 (–0.05, –0.68) ^*^	–0.11 (–0.47, 0.28)	–0.23 (–0.54, 0.18)	–0.21 (–0.53, 0.2)	–0.32 (–0.62, 0.12)
	**Thalamus**	**Caudate**	**Lenticular**	**PLIC**	**Cerebellum**
**Structures**					
Titmus Stereo Fly	0.58 (0.22, 0.8)^***^	0.27 (–0.08, 0.56)	0.29 (–0.07, 0.59)	0.12 (–0.23, 0.46)	0.04 (–0.39, 0.45)
Freiburg Visual Acuity	–0.37 (–0.66, 0.01)^*^	–0.09 (–0.45, 0.27)	–0.16 (–0.5, 0.21)	0.08 (–0.29, 0.4)	0.31 (–0.13, 0.63)
TVPS-4 visual discrimination	0.35 (–0.03, 0.65)^*^	0.07 (–0.29, 0.41)	0.18 (–0.19, 0.5)	–0.02 (–0.38, 0.34)	–0.26 (–0.57, 0.15)
TVPS-4 spatial relationships	0.3 (–0.08, 0.6)	0.2 (–0.16, 0.5)	0.24 (–0.07, 0.52)	0.01 (–0.34, 0.35)	–0.05 (–0.44, 0.36)
TVPS-4 form constancy	0.39 (0.01, 0.68)^*^	0.16 (–0.19, 0.48)	0.08 (–0.31, 0.44)	–0.01 (–0.34, 0.36)	–0.1 (–0.46, 0.28)
TVPS-4 visual figure–ground	0.47 (0.14, 0.69)^**^	0.21 (–0.12, 0.5)	0.29 (–0.09, 0.58)	0.09 (–0.22, 0.39)	–0.21 (–0.55, 0.21)
TVPS-4 visual closure	0.39 (0.03, 0.67)^*^	0.05 (–0.31, 0.38)	0.13 (–0.25, 0.48)	0.12 (–0.23, 0.45)	–0.1 (–0.45, 0.31)
Beery-VMI	0.49 (0.17, 0.74)^**^	0.3 (–0.02, 0.55)	0.39 (0.06, 0.64) ^*^	0.03 (–0.35, 0.38)	–0.14 (–0.5, 0.25)
FCVIQ	–0.45 (–0.07, –0.7)^**^	–0.07 (–0.37, 0.24)	–0.25 (–0.55, 0.11)	–0.28 (–0.58, 0.08)	0.02 (–0.34, 0.37)

Significant Spearman correlations between lesion-free volumes in the affected hemisphere and scores of clinical tests for vision scores were observed in most of the regions in the independent dataset (*n* = 34). Lower volume is associated with greater impairment across all tests. A positive correlation was found between volumes and the Titmus Stereo Fly, TVPS-4, and Beery-VMI tests, as lower scores indicated greater impairment. In contrast, a negative correlation was observed with the Freiburg Visual Acuity Test and FCVIQ, where higher scores reflected greater impairment. Spearman correlation coefficient and 95% confidence intervals.

WM, white matter; CGM, cortical gray matter; PLIC, posterior limb of the internal capsule; TVPS-4, Test of Visual Perceptual Skills, Fourth Edition; Beery-VMI, Beery Buktenica Test of Visual-Motor Integration, Sixth Edition; FCVIQ, flemish cerebral visual impairment questionnaire.

^*^p < 0.05.

^**^p < 0.01.

^***^p < 0.001.

## 4 Discussion

In this study, we developed, trained, and validated deep learning models tailored for quantitative brain measurements in children with uCP, enabling the automatic computation of regional lesion-free volumes. The models demonstrated good segmentation performance in patients with severe anatomical alterations and heterogeneous lesion appearances, both quantitatively and qualitatively. In addition, our findings support the hypothesis that the regional lesion-free volumes correlate with lesion severity and extent, as assessed by the sqMRI scale. Exploratory analyses of structure-function relationships revealed associations between regional lesion-free volumes and motor and vision outcomes.

While existing volumetric analysis tools such as childmetrix (Phan et al., 2021), FastSurfer (Henschel et al., 2020), and icobrain (Struyfs et al., 2020; Simarro et al., 2024) are widely used, they often encounter difficulties when segmenting brain images with large anatomical alterations, such as those observed in children with CP (Simarro et al., 2023). In response, we developed a workflow for generating silver ground truth labels, addressing the impracticality of manual annotations for large datasets. These labels allowed us to train deep learning models tailored to the uCP population, achieving good segmentation performance in patients with severe anatomical alterations and heterogeneous lesion appearances. This was demonstrated by a high overlap with silver ground truth in the testing dataset and a high approval rate for the segmentation quality provided by the pipeline, assessed by a neuroradiologist and a pediatric neurologist in the independent CP dataset. Additionally, the model's segmentation performance was validated on a fully independent dataset of children with congenital or acquired brain anatomy distortions. Despite the wide age range (1–17 years) and diverse lesion etiologies, including agenesis of the corpus callosum, posterior fossa malformations, malformations of cortical development, and severe brain distortions, the model achieved accurate results and generally outperformed the baseline U-Net model proposed for this dataset (Amorosino et al., 2022), particularly in key regions such as the deep GM.

Visual evaluation systems of MRI, such as sqMRI, are used in research and have been associated with cognition (Laporta-Hoyos et al., 2022), sensorimotor function (Fiori et al., 2015; Mailleux et al., 2017), and visual function (Tinelli et al., 2020). However, assessing the location and extent of brain damage in these children remains challenging, particularly in routine clinical settings (Fiori et al., 2014). Our study supported the hypothesis that automatically computed lesion-free volumes significantly correlate with visual evaluation of regional brain damage (i.e., sqMRI).

Volume loss in specific brain regions has been associated with declines in memory, verbal fluency, and visuospatial ability in cognitively normal older adults, reflecting domain-specific behavioral patterns corresponding to regional brain atrophy (Armstrong et al., 2020). In our exploratory analysis, lesion-free volume was associated with motor scores, including AHA and MACS, in subcortical structures such as the thalamus, caudate, and lenticular nucleus, as well as with the WM and CGM in the parietal, frontal, and temporal lobes. These findings align with previous literature, where lesions in the affected hemisphere of children with uCP were associated with motor hand function, including AHA (Fiori et al., 2015). In children with dyskinetic CP, global and parietal lobe lesions have been linked to poorer motor functioning, including the Gross Motor Function Classification System and MACS (Laporta-Hoyos et al., 2018). Additionally, in patients with traumatic brain injury, atrophy in the thalamus, putamen, and pallidum has been correlated with poorer bimanual performance (Gooijers et al., 2016). In this study, lower lesion-free PLIC volume was significantly associated with greater AHA impairment, but not significantly associated with MACS. In previous studies, visually-assessed PLIC lesions were associated with reduced sensorimotor performance (Fiori et al., 2015; Mailleux et al., 2017). This discrepancy may be due to challenges in accurately defining the PLIC anatomically automatically, as T1-weighted images lack sufficient contrast between the PLIC and surrounding WM tissue. Including diffusion-weighted imaging could enhance the accuracy of WM structure quantification, particularly for regions like the PLIC. Moreover, diffusion-weighted MRI could provide a complementary assessment of WM integrity to the volumetric analysis conducted in this study.

Lower total WM volume was associated with poorer visual functions and functional vision, including stereoacuity (Titmus Stereo Fly), visual acuity (Freiburg Visual Acuity), and motor-free visual-perceptual performance (TVPS-4). WM volumes in the frontal and temporal lobes were significantly correlated with TVPS-4 scores. In the frontal lobe, the Frontal Eye Field plays a key role in attentional and visual cognition (Vernet et al., 2014), and is structurally connected to the parietal cortex via the superior longitudinal fasciculus (Radwan et al., 2022). In the temporal lobe, the inferior longitudinal fasciculus is associated with object recognition deficits (Ortibus et al., 2012), further underscoring the importance of these regions in higher-order visual processing and potentially explaining the correlations we found. Interestingly, despite the role of the occipital lobe in primary visual processing, and previous research showing that occipital lobe lesion severity correlated with a general visual score (Tinelli et al., 2020), no significant correlation was found between occipital volume and visual function tests in this study. Further research is needed to clarify this finding. However, lesion-free WM volume in each lobe, including the occipital lobe, was positively associated with functional vision performance, as measured by the total FCVIQ score. This suggests that a global reduction in WM volume impacts functional vision limiting daily activities.

The study highlights the important role of the thalamus in motor and visual functions. These associations are well-documented in the literature: reorganization of thalamocortical projections has been linked to sensorimotor deficits (Tsao et al., 2015), and thalamic atrophy has been associated with abnormal visual development (Ricci et al., 2006). The lateral geniculate nucleus, part of the dorsal thalamus, is involved in transmitting visual information to the cerebral cortex (Usrey and Alitto, 2015). Thalamic lesions have shown strong correlations with visual scores (Tinelli et al., 2020). Similar associations were observed in children with dyskinetic CP, where visuospatial and visuoperceptive abilities were linked to lesions in the medial dorsal thalamus (Laporta-Hoyos et al., 2018). Additionally, reduced thalamic volume has been observed in infants with WM lesions and moderate to severe periventricular leukomalacia, with the reduction proportional to the extent of WM damage (Lin et al., 2001).

A limitation of this study is the use of silver ground truth for validation, which may artificially inflate performance metrics due to inherent similarities between the training and evaluation processes. To mitigate this, we performed an additional validation on an externally annotated dataset, as well as a visual assessment of the independent CP dataset.

Additionally, this study utilized a relatively small sample of children with uCP (35 for training and 54 for validation). This limitation, combined with the variability in lesion types and severities, may impact model performance in extreme cases (as shown in Figure 4A). Nonetheless, this study serves as a proof of concept, demonstrating the feasibility of tuning a deep learning model with a small sample size, which is often a constraint when recruiting pediatric patients, especially those with significant structural pathology. Furthermore, while the study focuses on unilateral CP, which is characterized by significant heterogeneity in clinical outcomes and lesion characteristics, the model's performance across other CP populations remains unexplored and warrants further investigation.

The proposed methodology provides a foundation for a standardized and automatic reporting system for MRI characteristics in uCP. This system could aid clinicians in identifying brain lesions and structural damage without relying on time-intensive visual classification methods, thereby enabling more consistent and objective measurements of brain structures in children with uCP. The association between lesion-free volumes and motor and visual outcomes underscores the potential of these measurements to detect functional impairments through brain imaging. Accounting for specific brain injury patterns in individual patients could facilitate personalized treatment strategies, potentially improving motor and visual outcomes.

## 5 Conclusion

The proposed pipeline for brain structure quantification can be used to study brain development and structure-function relations in children with uCP. By providing automated measurements of lesion-free structural volumes, this approach offers a comprehensive understanding of how brain structures correlate with a child's motor and visual functions. These findings support the potential use of structural MRI in personalized treatment planning selection.

## Data Availability

The datasets presented in this article are not readily available because restrictions in consent. Requests to access the datasets should be directed to jaime.simarro@icometrix.com.

## Footnotes

^1^ https://www.stereooptical.com/products/stereotests-color-tests/original-stereo-fly/
