# Supplementary material for: Automatic brain quantification in children with unilateral cerebral palsy
Source: Front Neurosci. 2025 Mar 10;19:1540480. doi: 10.3389/fnins.2025.1540480 (PMC11931148; doi:10.3389/fnins.2025.1540480)
Supplement: Supplementary file 1 [file Data_Sheet_1.pdf]

Supplementary Material: Automatic brain quantification in children with unilateral cerebral palsy

Acquisition protocol

MR images were acquired using a 3T Philips Achieva scanner with a 32-channel phased-array head coil. For the training and test set, high-resolution T1-weighted images (MPRAGE) were acquired with a spatial resolution of 1.2×0.98×0.98 mm^3 and TE/TR = 4.6/9.6 ms; T2-weighted fluid-attenuated inversion recovery (T2-FLAIR) images were acquired with a spatial resolution of 0.71×0.71×1.2 mm^3, TE/TR/TI = 415/4800/1650 ms. For the independent dataset, MPRAGE had a spatial resolution of 0.9×0.9×0.9 mm^3 and TE/TR = 4.2/9.1 ms, and T2-FLAIR images had a spatial resolution of 1×1×1 mm^3 and TE/TR/TI = 283/4800/1650 ms.

	Independent dataset
Titmus Stereo Fly	9.0 ± 3.6 (0.0 - 9.0)
Freiburg Visual Acuity	-0.1 ± 0.3 (-0.4 - 1.6)
TVPS-4 Visual Discrimination	-0.3 ± 1.2 (-2.7 - 1.7)
TVPS-4 Spatial Relationships	0.2 ± 1.2 (-3.0 - 1.3)
TVPS-4 Form Constancy	-0.3 ± 1.1 (-3.0 - 1.3)
TVPS-4 Visual Figure-Ground	-0.5 ± 1.1 (-3.0 - 1.3)
TVPS-4 Visual Closure	-0.7 ± 1.0 (-3.0 - 1.3)
Beery Visuomotor integration	-1.4 ± 1.0 (-3.7 - 0.0)[a]
FCVIQ	4.5 ± 7.5 (0 - 35)

Table S1. Clinical visual characteristics across the independent dataset. Mean ± standard deviation (min - max). TVPS-4 = Test of Visual Perceptual Skills, Fourth Edition,FCVIQ = Flemish Cerebral Visual Impairment Questionnaire. [a] Data available for 33 children.

	Dice similarity coefficient	Hausdorff distance 95th percentile
WM Parietal	0.97 (0.01)	0.78 (0.42)
WM Occipital	0.95 (0.01)	1.0 (0.0)
WM Frontal	0.98 (0.01)	0.44 (0.5)
WM Temporal	0.97 (0.01)	0.44 (0.5)
CGM Parietal	0.95 (0.01)	1.0 (0.0)
CGM Occipital	0.95 (0.01)	1.0 (0.0)
CGM Frontal	0.96 (0.01)	1.0 (0.0)
CGM Temporal	0.96 (0.01)	1.0 (0.0)
Lenticular	0.95 (0.03)	2.01 (2.84)
Thalamus	0.96 (0.02)	1.09 (0.29)
Caudate	0.95 (0.03)	1.12 (0.42)

Table S2. Quantitative validation of the segmentation model showed that the automatically computed brain structures achieved a high overlap with the silver ground truth in the test set. WM = White matter, GM = Gray matter. Mean (standard deviation).

Brain structures segmented by the structural segmentation model

1. Background

2. White matter background
3. Gray matter background
4. Cerebrospinal fluid background
5. Thalamus
6. Hippocampus
7. Putamen
8. Caudate
9. Pallidum
10. Midbrain
11. Pons
12. Medulla
13. Cerebellum
14. Upper lateral ventricles
15. Inferior lateral ventricles
16. Amygdala
17. Left CGM Frontal lobe
18. Right CGM Frontal lobe
19. Left CGM Parietal lobe
20. Right CGM Parietal lobe
21. Left CGM Occipital lobe
22. Right CGM Occipital lobe
23. CGM Temporal lobe
